# Thermal analysis of polyethylene + X% carbon nanotubes

**DOI:** 10.1186/s11671-016-1315-y

**Published:** 2016-02-24

**Authors:** Fedir Lozovyi, Kateryna Ivanenko, Sergii Nedilko, Sergiy Revo, Smail Hamamda

**Affiliations:** R&D Laboratory of Metal and Ceramics Physics, Taras Shevchenko National University of Kyiv, 64/13, Volodymyrska Street, 01601 Kyiv, Ukraine; Laboratory of Thermodynamics and Surface Treatment of Materials, University of Frères Mentouri Constantine 1, B.P. 325 Route Ain El Bey, Constantine, 25017 Algeria

**Keywords:** Nanomaterial, Polymer, Nanotube, Carbon, Thermal expansion, Anisotropy

## Abstract

The aim of this research is to study the influence of the multi-walled carbon nanotubes (MWCNTs) on the thermomechanical and structural properties of high-density polyethylene. Several, complementary experimental techniques were used, namely, dilatometry, differential scanning calorimetry (DSC), thermogravimetric analysis (TGA), Raman spectroscopy, and infrared (IR) spectroscopy. Dilatometry data showed that nanocomposites exhibit anisotropic behavior, and intensity of the anisotropy depends on the MWCNT concentration. The shapes of the dilatometric curves of the nanocomposites under study differ significantly for the radial and longitudinal directions of the samples. DSC results show that MWCNTs weekly influence calorimetry data, while Raman spectra show that the *I*_D_/*I*_G_ ratio decreases when MWCNT concentration increases. The IR spectra demonstrate improvement of the crystallinity of the samples as the content in MWCNTs rises.

## Background

Nowadays, carbon is especially attractive to researchers worldwide. If carbon was available, in the past, in one of the allotropic forms only, the number of these forms will have had increased of late. There were aroused intercalated graphites [1], expanded graphites [2], graphene [3], graphite foams [4], and fullerenes. After those, the carbon nanotubes came, and their appearance has revolutionized various areas of material science [5] and its applications, e.g., in medicine and environment protection.

Since the appearance of the carbon nanotubes (CNTs), several studies of their various characteristics have been conducted [6, 7]. The results taken by their authors relate to various physical quantities. The researchers independently reached the conclusion that CNTs introduced into various materials result in the improvement of the physico-chemical and thermodynamic properties of the made composites [8, 9], e.g., reinforcing polymers with nanotubes considerably alters their thermal and thermomechanical properties [10].

Wang et al. [11] have shown that the incorporation of nanotubes speeds up the crystallization of the polymers as nanotubes act as efficient agents of nucleation. Carbon nanotubes were also used for improving the thermal stability of the composites [12]. The thermal conductivity of nanotube-containing polymers offers new possibilities for their use instead of costly, resource-hungry metallic blocs of various applications, e.g., in power, electric engines and generators, and heat exchanger designs. The mentioned composites could be also a viable alternative to glass and aluminum in aeronautics. The substitution of those materials with nanotube-reinforcedpolymers is possible and cost effective thanks to the advantages that the latter ones offer. They are light, corrosion-resistant, and their elasticity and workability make them very competitive in several industrial applications [13–15].

## Methods

### Specimen preparation

The low-density polyethylene (LDPE) manufactured by “UfaOrgSynthesis” (Russia) with 918.5 ± 1.5 kg/m^3^ density and multi-walled carbon nanotubes (MWCNTs) were used for the sample preparation. The nanotubes were made by the CVD method in a rotating reactor [9]. Powder of the Al_2_O_3_–MoO_3_–Fe_2_O_3_ mixture was applied as catalyst. The propylene was just used as a source of carbon. The samples were prepared via multistage procedure. First, starting components, the LDPE and MWCNT powders, were mixed in the revolver-type rotational mixer. The rotation conditions were rotation rate 120 rev/min and rotation time 4 h. The isotropic mixtures were being taken after mixing. The mixture was loaded into a vacuum cylinder of extruder and heated up to 120 °C during 40 min at 10^3^ Pa pressure. After that, the mixture had been pushed through a die. The made cylinders of the composites were of 3 mm in diameter, and their high was near 5 mm. When measurements were carried on, the direction along the axis of the cylinder was observed as “longitudinal”, while the direction along a diameter of the cylinder had been denoted as “radial.” Two samples from each type were used for the dilatometric tests; one was placed along the longitudinal direction and the other along the radial direction.

The starting mixtures of the MWCNTs’ different fractions (0, 0.5, 1.0, and 2.5 %) were taken, so four types of the samples were prepared for measurements.

### Equipment used

- A NETZSCH 402C dilatometer (NETZSCH, Selb, Germany) with 3 % accuracy was used in the study. The heating rate was near 10 °C/min. The thermal expansion coefficient was measured in the temperature range from 25 to 110 °C.
- Differential scanning calorimetry (DSC) and thermogravimetric analysis (TGA) tests were conducted using a Jupiter STA 449 F3 calorimeter by NETZSCH (NETZSCH, Selb, Germany). The same heating rate as in the dilatometric measurements was used.
- The infrared absorption and Raman spectra were measured using a Jasco FT/130 IR-6300 (Jasco Analytical Instruments, Easton, MD, USA) and the Bruker SENTERRA (Bruker, Billerica, MA, USA), respectively.

## Results and discussion

The results obtained are illustrated in the figures below.

**Fig. 1:**
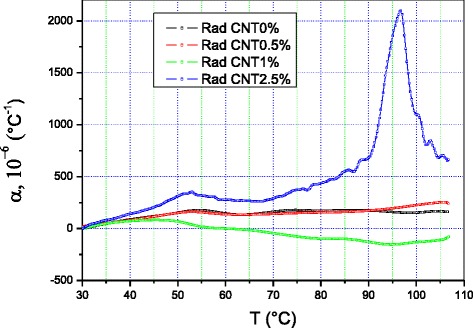
Thermal expansion coefficient of PE + X % MCNT nanocomposites along radial direction

**Fig. 2:**
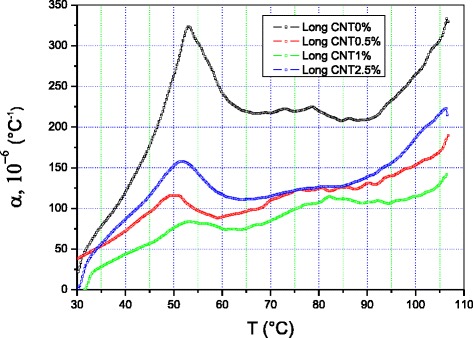
Thermal expansion coefficient of PE + X % MCNT nanocomposites along longitudinal direction

Figures 1 and 2 illustrate the dilatometric behavior of various LDPE-based nanocomposites with different concentrations of MWCNTs.

The thermal expansion coefficients of the samples measured along the radial direction, *α*_R_(T), and the longitudinal direction, *α*_L_(T), vary differently with temperature. The difference in variation is significant. This implies that the material is strongly anisotropic. The two curves exhibit a dilatometric singularity around 50–55 °C. The peak in the curve measured along the longitudinal direction is twice as intense as the peak measured along the radial direction, which is 30 °C wide. The curve *α*_L_(T) levels off between 65 and 90 °C; then, *α*_L_ abruptly resumes its increase with temperature until it reaches 330 °C^−1^ around 105 °C, while *α*_R_(T) is not higher than 150 °C^−1^ at this temperature. The ratio *α*_L_(T)/*α*_R_(T) is significantly larger than 100 %.

The MWCNTs also influence the dilatometric behavior of the LDPE composites. At 0.5 % MWCNTs, we observe a decrease in the intensity of the anisotropy and a reversal of roles. Starting from 35 °C, *α*_R_(T) is larger than *α*_L_(T) over the rest of the temperature range applied. The singularity on the *α*_L_(T) curve moves to lower temperatures, and its intensity exhibits a larger-than-threefold decrease in intensity compared to *α*_L_(T) in the pure polymer. The temperature at which *α*_R_(T) singularity appears did not change significantly, but its intensity decreased a little and its shape changed; it is less spread as compared to the *α*_R_(T) of the pure sample. The two curves have the same shape and develop similarly starting from 65 °C.

When the concentration reaches 2.5 % MWCNTs, the shapes of the *α*_R_(T) and *α*_L_(T) curves change significantly. We note that they do not include the same number of peaks. The first two, situated at around 50 °C, have the same shape and different intensities. The peak on the *α*_R_(T) curve is practically twice as intense as the one on the *α*_L_(T) curve. At 95 °C, there is a second dilatometric peak which appears only on the *α*_R_(T) curve. Its intensity is four times greater than that of the peak at lower temperatures.

Comparison of the *α*_L_(T) curves obtained for the samples with different MWCNT concentrations shows that they basically have the same shape, which confirms the view that they have the same origin. All of them exhibit a singularity at around 50–55 °C. Its intensity, however, varies with the MWCNT concentration. The peak intensities vary between 80 and 325 °C^−1^. It is 80 °C^−1^ when there is 1 % MWCNT in the polymer. Halving this concentration to 0.5 % increases the peak intensity to 120 °C^−1^. The intensity is 160 °C^−1^ at 2.5 % concentration, while it reaches more than 320 °C^−1^ for the pure polymer. Starting from 35 °C, the ascending order of the thermal expansion coefficients is *α*_L_(1 %) < *α*_L_(0.5 %) < *α*_L_(2.5 %) < *α*_L_(0 %). At 100 °C, the ratios are *α*_L_(0 %)/*α*_L_(0.5 %) = 180 %, *α*_L_(0 %)/*α*_L_(1 %) = 225 %, and *α*_L_(0 %)/*α*_L_(2.5 %) = 142 %.

We had also observed that the introduction of the nanotubes significantly decreased the thermal expansion coefficient of the polymer along the longitudinal direction. We observed that, first, the *α*_L_(T) curve at 0.5 % concentration is comprised between the *α*_L_(T) curves at 2.5 and 1 % concentrations over the whole temperature range under study, and second, thermal expansion is at its weakest for the sample with 1 % MWCNT concentration. Thus, the addition of 1 % MWCNTs may be responsible for the increase of interaction forces within the material.

The samples containing 0, 0.5, and 1 % MWCNTs have *α*_R_(T) curves that are basically undistinguishable below 43 °C. Beyond this temperature, the *α*_R_(T) curve of the sample with 1 % MWCNTs differs from the other two by decreasing over the rest of the temperature range, while *α*_R_(0 %) and *α*_R_(0.5 %) still overlap up to 90 °C. Starting from 93 °C, *α*_R_(0 %) becomes smaller than *α*_R_(0.5 %). At 105 °C, this decrease reaches 40 %.

When the concentration in MWCNTs is 1 %, the made nanomaterial expands the least if compared with the other three. When the concentration reaches 2.5 %, the coefficient of thermal expansion is distinctly different from the other three. The *α*_R_(2.5 %) is larger than the others over the whole temperature range. The shape of the dilatometric curve of this sample is not similar to the other three. It exhibits two dilatometric singularities. The first peak, observed on all the curves and appearing at the same temperature, has a higher intensity than the other three. The second very intensive singularity, with a thermal expansion coefficient larger than 2000 °C^−1^, appears at 96 °C and is only visible on the curve corresponding to the material with 2.5 % MWCNTs.

We notice that, regardless of the direction of the measurement, the thermal expansion coefficient is the smallest for the nanocomposite with 1 % MWCNTs. Thus, the introduction of this fraction of MWCNTs into the LDPE material strongly influences the dilatometric behavior of the nanocomposite, resulting in an important decrease in its expandability. This is the result of a strengthening of the interaction forces that is conducive to better stability of the nanocomposite material.

So, we point the decrease of the thermal expansion coefficient for both longitudinal and transverse directions of the samples, if MWCNTs are added to the LDPE at content below or equal to 1 %. If the fraction of the MWCNTs is higher than 1 %, the reverse dependences of the α_R_ and α_L_ on the MWCNT content occur. The rates of the α_R_ and α_L_ increase are different (Figs. 1 and 2). As a result, α_R_(T) and α_L_ (T) curves for 0.5 % MWCNT fractions are located between the curves measured for 1 and 2.5 %. The described behavior is related with the abovementioned anisotropy of the samples, and the procedure of the sample preparation, via axial pressing, is a reason of the anisotropy.

**Fig. 3:**
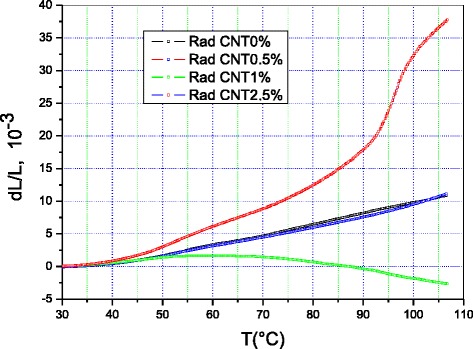
Relative dimensional variation (Δ*L*/*L*) of polyethylene containing 0, 0.5, 1, and 2.5 % multi-walled carbon nanotubes along the radial direction

**Fig. 4:**
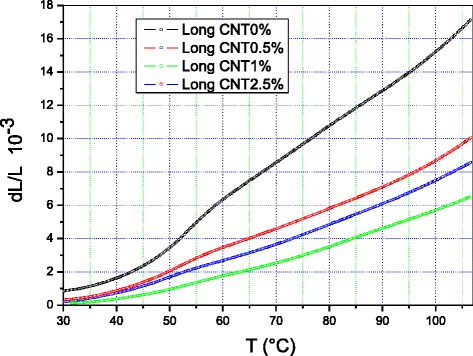
Relative dimensional variation (Δ*L*/*L*) of polyethylene containing 0, 0.5, 1, and 2.5 % multi-walled carbon nanotubes along the longitudinal direction

Figures 3 and 4 show the relative variation in length Δ*L*/*L* of the polyethylene containing 0, 0.5, 1, and 2.5 % MWCNTs measured along the radial and longitudinal directions, respectively. The dimensional variation of the four samples depends heavily on the direction of measurement.

The relative variation in dimension of PE + 1 % MWCNTs along the longitudinal direction is the smallest over the whole temperature range of the study. Similarly, the same nanocomposite exhibits the smallest dimensional variation along the radial direction of the four samples.

**Fig. 5:**
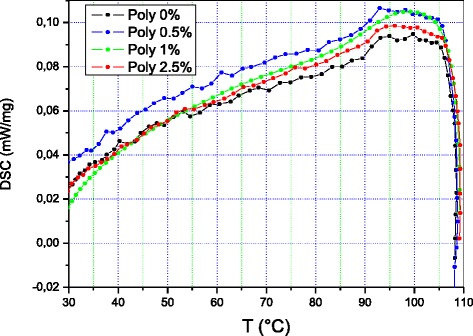
Differential scanning calorimetric diagram of PE + X % MCNT nanocomposites

Examination of the calorimetric curves shown in Fig. 5 demonstrates that the curve of the polyethylene sample containing 0.5 % MWCNTs is above the other three up to a temperature slightly above 90 °C, while the curves of the other three samples overlap.

At around 95 °C, each of the four curves exhibits a calorimetric singularity 15 °C which is wider that peaks at about 100 °C. Starting from 105 °C, the curves become undistinguishable and decrease abruptly.

**Fig. 6:**
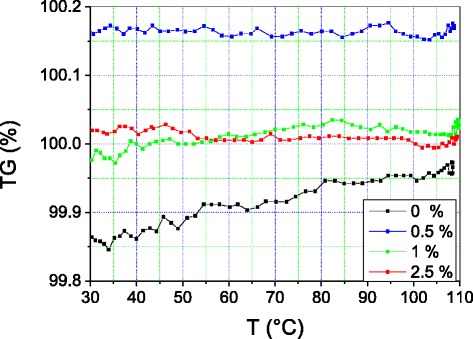
Thermogravimetry of PE + X % MCNT nanocomposites

The TGA curves of the samples containing 1 and 2.5 % MWCNTs overlap and have reasonably equal intensities (Fig. 6).

The curve of the sample containing 0.5 % MWCNTs is significantly more intense, over the whole temperature range, than those that the other two nanocomposites reveal. The difference between the TGA curve of PE + 0.5 % MWCNTs and the others remains reasonably constant over the whole temperature range. We also notice that, regardless of the concentration in MWCNTs, the TGA curves of the nanocomposites are all above the curve of the pure polymer. Starting from 100 °C, the TGA curve of the pure material overlaps with those of the materials containing 1 and 2.5 % MWCNTs.

**Fig. 7:**
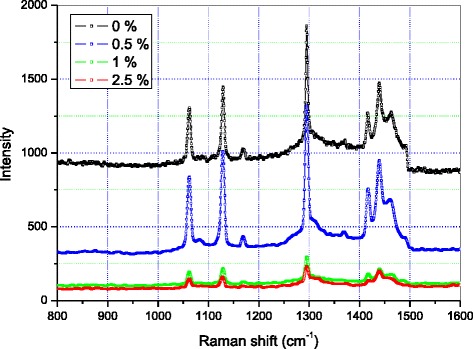
Raman spectra of PE + X % MCNT nanocomposites

Figure 7 below shows the Raman spectra of the samples.

The intensity of the various peaks observed on the four spectra strongly varies with the nanotube concentration. The spectra of the pure sample and the one containing 0.5 % MWCNTs can be superimposed. The peaks on the PE + 0.5 % MWCNT spectrum are more intense than those of the pure PE. The spectra of the samples containing 1 and 2.5 % MWCNTs are similar in shape and can be superimposed. The two spectra exhibit the same number of peaks, and these appear at the same frequencies. However, all the peak intensities are clearly higher in the spectrum for PE + 1 % MWCNTs than the corresponding intensities of the PE + 2.5 % MWCNT spectrum. The Raman intensity of the PE + 2.5 % MWCNTs is lower than that of the other three.

We evaluated the intensity of the *I*_D_ and *I*_G_ Raman peaks. It is known that the *I*_D_/*I*_G_ ratio reflects disorder and a number of defects in the solid networks. The introduction of 0.5 % MWCNTs results in an *I*_D_/*I*_G_ ratio of about 180 %. The ratio (*I*_D_/*I*_G_ ≈ 120 %) decreases for the sample at 1 % concentration of MWCNTs. Addition of more MWCNTs further decreases the intensity of all the peaks in the spectrum; the ratio reaches *I*_D_/*I*_G_ ≈ 105 %. Thus, the increase in the concentration of MWCNTs results in the decrease in the peak intensity and the *I*_D_/*I*_G_ ratio.

**Fig. 8:**
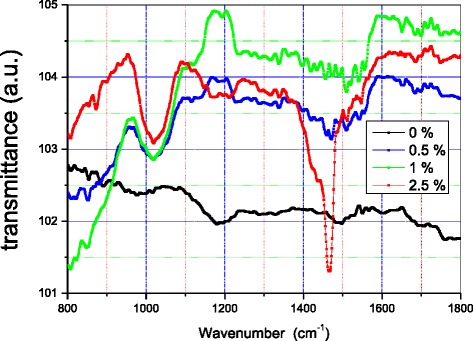
Infrared spectra of PE + X % MCNT nanocomposites

Figure 8 shows the infrared spectra of the three nanomaterials and that of pure polyethylene.

Spectral analysis using Fourier transform infrared (FTIR) shows a significant increase in the intensity of the spectra resulting from the introduction of carbon nanotubes into polyethylene. The spectra of the three samples containing nanotubes changed shape and became more intense. The spectrum of the PE + 1 % MWCNT sample is the most intense. At high wave number (*k*) values, the FTIR spectra of the three nanocomposites have the same shape, but their intensities depend on the concentration of MWCNTs. Increase in the concentration from 0.5 to 1 % MWCNTs results in a more intense spectrum. Further increase in the concentration of MWCNTs, from 1 to 2.5 %, however, results in the opposite effect. The FTIR spectrum of PE + 2.5 % MWCNTs decreases in intensity. The bands appearing after polyethylene-doping change the shape, and the changes depend on the quantity of the added carbon nanotubes. The bands become narrower and tend to morph into peaks. At 2.5 % concentration in MWCNTs, we observe intense peaks, which are characteristics for good crystallinity of polymers. Thus, the increase of the MWCNT concentration improves the crystallinity of the polymer matrix.

The different experimental techniques used are complementary and confirm the positive effect of the of multi-walled carbon nanotube introduction into a polymer matrix as they improve the thermomechanical and structural properties of the nanocomposites (see also, e.g., [16–18]).

## Conclusions

Dilatometric tests demonstrate that the addition of MWCNTs decreases the anisotropy of the nanocomposite and strengthens the interactions inside it. Spectral analysis confirms the improvement in mechanical properties. Raman spectroscopy shows that the *I*_D_/*I*_G_ ratio decreases with the increase in the MWCNT concentration, which is probably related to the decrease in the relative number of defects. FTIR spectroscopy shows that the crystallinity is improved with the increase in the MWCNT concentration. The TGA curves of the nanocomposites exhibit practically negligible slopes if compared to pure polyethylene. The calorimetric behavior is reasonably the same for all four samples studied.

A weak expansion, a practically constant thermogravimetry, an *I*_D_/*I*_G_ ratio which decreases with the increase in concentration, and a crystallinity that improves when the quantity of carbon nanotubes added increases point to a positive effect of multi-walled carbon nanotubes when they are incorporated into low-density polyethylene.
